# MEYELens: An affordable, open-source, 3D-printable eyewear platform for pupillometry and gaze tracking

**DOI:** 10.3758/s13428-026-03157-z

**Published:** 2026-08-21

**Authors:** Giacomo Vecchieschi, Letizia Ingenito, Alessandro Benedetto, Caterina Luciani, Fabio Carrara, Giovanni Cioni, Andrea Guzzetta, Tommaso Pizzorusso, Laura Baroncelli, Raffaele M. Mazziotti

**Affiliations:** 1 Department of Developmental Neuroscience, IRCCS Stella Maris Foundation, Pisa, Italy; 2 Department of Translational Research and New Technologies in Medicine and Surgery, University of Pisa, Pisa, Italy; 3 Department of Neuroscience, Psychology, Pharmacology, and Child Health, University of Florence, viale Gaetano Pieraccini, 6, 50139 Florence, Italy; 4 BIO@SNS Lab, Scuola Normale Superiore, Pisa, Italy; 5 Institute of Information Science and Technologies, National Research Council, Pisa, Italy; 6 Institute of Neuroscience, National Research Council, Pisa, Italy

**Keywords:** Pupillometry, Gaze tracking, Eye tracking, Wearable eye-tracking system

## Abstract

Pupillometry and gaze tracking provide sensitive, noninvasive indices of autonomic function and cognitive state and are increasingly employed in neuroscience and clinical research. However, commonly used systems remain costly and often constrain hardware customization and workflow transparency. MEYELens is presented as an open-source, fully 3D-printable wearable system for low-cost pupillometry and gaze tracking using readily available components. The design is modular and mechanically adjustable to accommodate different users and experimental requirements. An open pipeline is provided for data acquisition and for both offline and online analysis. Feasibility is demonstrated across frequency-based and task-evoked pupillary paradigms, and gaze mapping is shown to operate in both screen-based and naturalistic settings. By combining affordability, modular hardware, and open software, MEYELens reduces barriers to reliable eye-based measurement and supports methodological development in resource-constrained research contexts.

**Supplementary Information:**

The online version contains supplementary material available at https://doi.org/10.3758/s13428-026-03157-z.

## Introduction

Pupil diameter is not determined exclusively by the optical properties of incoming light or by visual accommodation demands; it also reflects the influence of central neuromodulatory signals associated with arousal, cognitive load, and affective state, thereby providing an indirect measure of brain state (Mathôt, 2018). As a result, pupillometry, the measurement of pupil size over time, has emerged as a valuable tool for identifying biomarkers in both preclinical and clinical contexts (Artoni et al., 2020; Martínez-Palacios et al., 2024; Maxin et al., 2025; Moore et al., 2018; Shah et al., 2025; Teixeira et al., 2021). Clinically, pupillometry has been applied across a wide range of contexts, including the monitoring of intracranial pressure (Martínez-Palacios et al., 2024), assessment of traumatic brain injury (Teixeira et al., 2021), and characterization of stroke-related alterations (Maxin et al., 2025), as well as the evaluation of the pharmacodynamic effects of centrally acting drugs (McKay & Larson, 2021). It has also gained particular relevance in the study of neurodevelopmental disorders. These include common conditions such as autism spectrum disorder (de Vries et al., 2021; Nyström et al., 2018; Pomè et al., 2020; Turi et al., 2018), attention-deficit/hyperactivity disorder (Das & Khanna, 2021; Duque-Chica et al., 2025), Tourette syndrome (Tharp et al., 2015), specific language impairment (Lum et al., 2017), Down syndrome (Angulo-Chavira et al., 2017), as well as rare syndromes including Rett syndrome (Artoni et al., 2020) and CDKL5 deficiency disorder (Viglione et al., 2022), where pupillometry is emerging as a candidate source of functional biomarkers for monitoring disease progression and the efficacy of treatments.

In cognitive neuroscience, pupillometry has been widely employed to investigate working memory (Mathôt & Vilotijević, 2022; van der Wel & van Steenbergen, 2018), lexical access (Rojas et al., 2024), and ocular dominance plasticity (Acquafredda & Binda, 2024), among a wide range of other cognitive and perceptual processes (Castellotti et al., 2025; Mathôt & Vilotijević, 2022).

In parallel, gaze tracking has become a widely studied approach for both diagnostic (Moore et al., 2018; Shah et al., 2025) and therapeutic applications (Carelli et al., 2022). Clinically, gaze tracking has been investigated for brain-injury assessment, where altered fixation, smooth pursuit, and saccadic metrics are frequently reported and discussed as potential objective markers (Cade & Turnbull, 2024; Hunfalvay et al., 2021). Moreover, gaze tracking is also being explored for stroke diagnosis and assessment (Hassan et al., 2025; Zhang et al., 2025). In neurodevelopmental disorders, atypical gaze allocation and attention patterns have been evaluated for ASD screening, while portable approaches have also been proposed for ADHD characterization (Tecar et al., 2025; Yoo et al., 2024).

Beyond clinical neuroscience, gaze tracking plays a central role in psychophysics and neuroeconomics research, providing insights into, among others, visual attention and perceptual processing (Lima & Ventura, 2023), as well as decision-making and choice dynamics (Krajbich & Rangel, 2011; Ting & Gluth, 2024; Wedel et al., 2023).

Both pupillometry and gaze tracking are increasingly leveraged in the design of human--computer interaction systems, supporting adaptive interfaces and user-state monitoring (Mathôt et al., 2016; Onyemauche et al., 2021).

Despite their wide applicability, current commercial solutions for pupillometry and gaze tracking are often relatively expensive and lack the flexibility required to tailor hardware and software configurations to specific experimental or clinical needs (Casas & Chandrasekaran, 2019; Toivanen et al., 2017; Zandi et al., 2021). These limitations reduce accessibility, constrain methodological innovation, and largely restrict their use to controlled laboratory or clinical settings. Cost-effective, open-source solutions have been proposed (Chacko et al., 2025; de Souza et al., 2013; Picanço & Tonneau, 2018; Zandi et al., 2021), but they often require substantial programming or electronic expertise, or lack validation across established experimental paradigms, thereby limiting their deployment in unsupervised or home-based contexts, such as data collection performed by non-specialist users including parents or caregivers.

To address these limitations, we developed MEYELens, a 3D-printable wearable system for pupillometry and gaze tracking that, when combined with readily available off-the-shelf components and low-cost cameras, enables an affordable and easily assembled solution. The design is modular and mechanically flexible, allowing adjustment across multiple degrees of freedom and customization for specific experimental requirements. The open-source software further supports extensibility and integration across diverse research contexts.

## Materials and methods

### 3D-printed headset

**Fig. 1:**
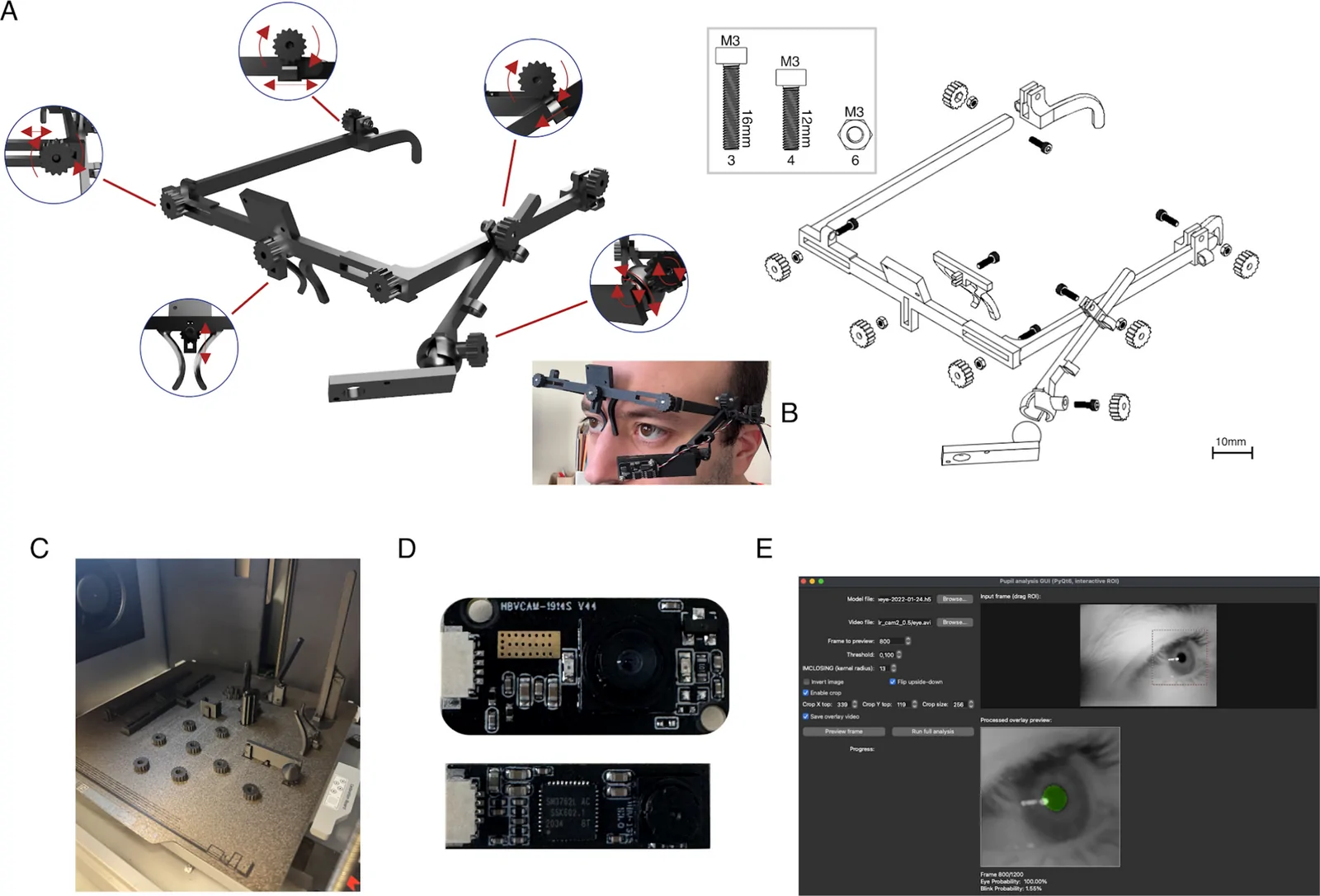
MEYELens hardware and software overview. **A** Render of the 3D-printed glasses frame showing the adjustable joints and mounting points (*highlighted in the insets*) and an example of the device worn on the face. **B** Exploded assembly schematic of the frame, illustrating the main printed parts and the required M3 fasteners (screws and nuts) used to assemble the structure and supports. **C **3D-printed components on the bed of the 3D printer. **D** Camera modules used in the system (GC0307 top and GC0308 bottom). **E** The MEYELens GUI used for offline processing, showing interactive ROI selection on the eye image and an overlay preview of pupil segmentation

The headset hardware (Fig. 1A, B) was designed in Autodesk Fusion (Autodesk, 2025) and exported in stereolithography (STL) format. The 3D models were sliced using Bambu Studio (Bambu Lab, 2025) and printed on a Bambu Lab X1-Carbon 3D printer using Bambu Lab Matte Black Polylactic Acid (PLA) filament. Printing parameters included a 50% gyroid infill, 0.2-mm layer height, two wall loops, enabled supports, and a 0.4-mm nozzle. Print speeds ranged from 50 to 105 mm/s for the first layer and 200–300 mm/s for subsequent layers. The print required approximately 55 g of filament and took about 3 h to complete on the printer used. Using a reduced infill (15%) decreases print time to approximately 2.5 h, at the expense of mechanical robustness. It can be printed all at once (Fig. 1C). The STL file for the model is provided in the supplementary materials. In addition, the 3D Manufacturing Format (.3mf) file is included; this preserves the full print setup (orientation, supports, and slicer settings), enabling direct printing without further configuration.

The system supports multiple hardware configurations. It can be fitted with a single eye-facing camera for pupillometry and chin-rested gaze tracking, or with dual cameras comprising an eye-facing camera and a world-facing camera for naturalistic gaze tracking.

### Cameras

Two types of camera modules were used. The first was a GC0307 camera (Fig. 1D top), with a resolution of 640 × 480 pixels and a nominal frame rate of 30 Hz. This model was originally equipped with an infrared-cut filter, which was manually removed under a microscope to allow the infrared illumination required for pupillometry. The camera was paired with an external 96-LED infrared illuminator. The second type consisted of GC0308 cameras (Fig. 1D bottom), also with a resolution of 640 × 480 pixels and a nominal frame rate of 30 Hz. These cameras include built-in infrared LEDs and are available with different fields of view. In this work, a 50° lens was used for eye-facing recordings and an 80° lens for world-facing recordings.

During preliminary testing, although the cameras occasionally reached their nominal frame rate, acquisition frequently dropped to approximately 20 frames per second (Supplementary Figure 1). To ensure stable and consistent recordings, all cameras were therefore operated at a maximum frame rate of 20 Hz. In addition, the built-in infrared LEDs of the GC0308 cameras sometimes produced insufficient or spatially uneven pupil illumination. When required, this was addressed by mounting a custom infrared LED array on the camera support (Supplementary Figure 2).

### Assembly

Assembly was carried out as follows. Three M3 × 16 mm screws were installed in the frontal frame of the headset to attach the temple arms and nose support to the main frontal body. An additional four M3 × 12 mm screws were used: two to attach the ear supports, one to connect the camera arm, and one to secure the ball bearing supporting the camera. All M3 screws, except the one anchoring the ball bearing, were secured with M3 nuts (six in total). This is schematized in Fig. 1B.

Cameras were mounted to the headset using two M2 × 10 mm screws and corresponding M2 nuts. For the GC0307 camera, the module was fixed to the support using adhesive tape.

To ensure mechanical stability, screw heads intended to remain fixed and the nut supports were secured using UV light-curing adhesive (Visbella, 797434003843). USB cables were routed through dedicated cable channels and connected to either a standard USB extension cable, a dual-headed USB extension cable, or a USB-C hub, depending on the number of cameras required for a given configuration.

### Data acquisition and processing

Camera recordings were acquired using custom scripts written in Python (3.8.18) and OpenCV version 4.11.0.86 (Bradski, 2000). Visual and auditory stimuli were generated using PsychoPy version 2024.2.1 (Peirce et al., 2019). AprilTags (Olson, 2011) were generated and detected using the Aruco library of OpenCV (Garrido-Jurado et al., 2014). When experimental paradigms required two video streams for gaze tracking, the dual-camera configuration was used; when only the eye-facing camera video was required, the single-camera configuration was employed. Both online and offline pupil segmentation were performed using MEYE (Mazziotti et al., 2021), a deep learning tool that segments the pupil from eye images and classifies each frame as containing either an eye or a blink. For each frame, the pipeline extracted pupil area, centroid coordinates (*x* and *y*), and the probability of eye or blink presence, which were stored as time-stamped rows in a comma-separated values (CSV) file. Frames classified as blinks were removed, and the corresponding samples were linearly interpolated. Segmentation parameters were set as follows: *threshold* = 0.1, *imclosing* = 13, and invertimage = False. Parameters were fine-tuned when necessary to improve segmentation accuracy. To facilitate reuse and reproducibility, the full pupil-processing workflow was released as an open-source Python package, *meyelens*, distributed via the Python Package Index (PyPI) and installable using a pip command. The package provides both an application programming interface (API) and a graphical user interface (GUI) for offline analysis (Fig. 1E). The GUI, implemented in PyQt6 (version 6.10.1), allows users to select the segmentation model and input video, interactively define the eye region of interest by drawing a bounding box on a preview frame, and optionally enable cropping by specifying ROI coordinates and crop size. Key preprocessing and segmentation parameters, including threshold, morphological closing kernel radius, image inversion, and vertical flipping, can be adjusted with immediate visual feedback via an overlay showing the detected pupil mask and per-frame eye or blink probabilities. Once parameters are finalized, the full analysis mode processes the entire recording and exports the time-stamped per-frame CSV file and, optionally, a quality-control overlay video. For gaze tracking, calibration was performed using an AprilTag (tag family: tag36h11, ID: 15) presented sequentially at five screen locations: the four corners and the center. This tag was selected because its central region provides a clear fixation point. Each tag presentation was preceded by a white fixation cross displayed for 1 s to cue gaze. Tags measured 3 × 3 cm and were displayed for 5 s each. Participants were instructed to maintain fixation on the center of the tag throughout calibration. Following calibration, the videos from the two cameras were processed. The eye-facing camera video was analyzed using the MEYE pipeline (Mazziotti et al., 2021) to extract pupil centroid coordinates for each frame. The world-facing camera video was processed using OpenCV to track the spatial position of the calibration AprilTag. A custom regression model implemented in scikit-learn (version 1.6.1; Buitinck et al., 2013) was used to map two-dimensional pupil center coordinates to corresponding two-dimensional world-facing camera coordinates. Input features were first standardized using the StandardScaler function, after which a second-degree polynomial feature expansion was applied using *PolynomialFeatures*, excluding the bias term. The transformed features were used to fit a *LinearRegression* model that predicted standardized world-facing camera coordinates. Predicted values were then inverse-transformed to obtain gaze positions in pixel units and appended to the original CSV file. For applications requiring a simplified experimental setup, gaze tracking was performed using a single eye-facing camera in combination with a chin rest to stabilize head position and maintain a fixed spatial relationship between the eye and the display. In this configuration, the screen coordinates associated with each calibration point were obtained directly from the stimulus presentation script, thereby removing the need for a world-facing camera. All subsequent processing steps, including pupil detection, feature transformation, and regression mapping, were identical to those used in the dual-camera configuration. Gaze-calibration generalization was evaluated using the single-camera, chin-rested configuration. Stimuli were presented on an Acer KG272U G monitor with a native resolution of 2560 × 1440 pixels, within a PsychoPy window subtending 40.9 × 23.7° of visual angle at a viewing distance of 50 cm. Calibration was performed using five positions: one at the center of the stimulus window and four near the corners, corresponding to approximately ± 18.6° horizontally and ± 9.8° vertically relative to the window center. After calibration, the fitted model was tested on 20 pseudorandom validation positions within the same window. These validation positions were not used for model fitting. For each validation position, 60 processed gaze samples were obtained, yielding a total of 1200 held-out validation samples. Angular error was defined as the Euclidean distance between the predicted gaze position and the true validation target position. Mean, median, and root-mean-square errors were then computed across sample-level angular errors.

### Pupillary light reflex (PLR)

Each trial began with a 5-s pre-stimulus period during which a black screen (0 cd/m^2^) with a central red fixation dot was displayed. This was followed by the presentation of a centrally positioned square stimulus (8 × 8 cm) for 0.5 s. The sequence was repeated for a total of ten trials per session. The experiment was conducted across four separate runs, each using a different stimulus luminance level (4.68, 23.70, 61.21, and 120.00 cd/m^2^), spanning a range from near black to white. Data were collected from a single participant (male, 29 years old).

### Pupil frequency tagging (PFT)

Each trial began with a 5-s pre-stimulus period during which a uniform gray screen (23.70 cd/m^2^) with a central red fixation dot was displayed, followed by a 20-s stimulation period. During stimulation, a centrally positioned square stimulus (8 × 8 cm) alternated between white (120 cd/m^2^) and black (0 cd/m^2^) at a fixed temporal frequency. The following flicker frequencies were tested: 1, 1.1111, 1.25, 1.4286, 1.6667, 2, 2.5, 3.3333, and 5 Hz. Frequencies were selected to be compatible with the camera sampling rate of 20 Hz, such that each flicker cycle consisted of an integer number of video frames. This constraint ensured consistent phase durations across trials and minimized temporal variability. The experiment was conducted with three male participants (ages 26, 29, and 41 years).

### Oddball paradigm

Participants viewed a uniform gray screen (23.70 cd/m^2^) displaying a central black fixation cross and wore headphones (Trust GXT 433). Auditory stimuli were generated as pure sine-wave tones using the PsychoPy sound engine. Three tone types were presented: a standard tone (440 Hz; 90% of trials), an oddball target tone (880 Hz; 5% of trials), and a distractor tone (220 Hz; 5% of trials). All tones had a duration of 100 ms and were presented with an interstimulus interval of 500 ms. Trial order was randomized for each participant. Participants were instructed to press the spacebar upon detection of the oddball tone and to withhold responses to the distractor tone. The session terminated after each participant had completed 20 oddball and 20 distractor trials. Five participants were tested (mean age = 28 ± 6.36 years).

### Parallel PLR

The experimental paradigm was identical to the pupillary light reflex protocol described above, with the exception that recordings were acquired simultaneously from two camera units. Stimulus luminance levels, as measured in OpenCV intensity units and expressed in cd/m^2^, were 0.11, 1.42, 4.68, and 10.29, spanning a range from black to gray. The experiment was conducted with two male participants (ages 29 and 41 years).

### Comparison with a commercial eye tracker

The experiment was conducted using the single-camera configuration in parallel with a commercial eye-tracking system (Gazepoint GP3) for comparison. The Gazepoint GP3 was operated at its nominal acquisition frequency of 60 Hz. One participant (male, 35 years old) took part in the experiment. The participant was seated in front of a monitor with head position stabilized using a chin rest. Eye position and pupil diameter were recorded using MATLAB via the Gazepoint-MATLAB toolbox. In parallel, the synchronized eye-facing camera video stream was recorded using the *VideoCapture* function of Psychtoolbox (Brainard, 1997; Pelli, 1997). Recordings from MEYELens and the GP3 were acquired simultaneously during the same experimental trials. To prevent potential infrared interference between systems, the MEYELens infrared illumination was disabled during this comparison; infrared illumination was provided exclusively by the GP3, which also provided sufficient illumination for the MEYELens eye-facing camera. Visual stimuli were generated in Psychtoolbox, and the recorded camera stream was subsequently processed using the MEYE pipeline.

For the PFT condition, a centrally positioned circular stimulus was presented with luminance sinusoidally modulated at 1 Hz. Trials lasted 8 s and were repeated ten times. Each display refresh was time-stamped, and frame-level markers were sent to the GP3 at every refresh, allowing precise offline alignment of pupil samples, stimulus luminance, and camera frames.

For the smooth pursuit condition, the same acquisition configuration was used, but the stimulus consisted of a small red target moving horizontally back and forth, with position modulated over time by a sinusoidal function at 0.2 Hz (± 15° visual angle). Eye position samples and synchronized camera frames were recorded on a per-frame basis, with timing markers sent to the GP3 to enable frame-accurate comparison between recorded gaze position and target motion.

### Naturalistic gaze-tracking

Following camera calibration, which was performed by presenting the calibration sequence on a separate computer, participants (*N* = 3) were asked to stand facing a test area containing four boxes, each labeled with a unique AprilTag (family: 36h11, IDs: 2; 3; 4; 5). A smartphone (iPhone 15 Pro; volume set to 50%; ringtone: “By The Seaside”) was concealed behind one of the boxes. During each trial, the phone was triggered to ring, and participants were instructed to locate the sound source and verbally report the position of the phone after the ringing had stopped.

### Computer and display

Visual stimulation and video acquisition for the pupillary light reflex (PLR), pupil frequency tagging (PFT), parallel PLR, and commercial eye-tracker comparison paradigms were conducted on an Apple MacBook Pro M3 Pro (11-core CPU, 14-core GPU, 18 GB unified memory; display resolution 1512 × 982 pixels). ProMotion and True Tone features were disabled to ensure consistent visual presentation. The display was calibrated using a Datacolor SpyderX Pro colorimeter (gamma: 2.18, at 50% luminance).

The oddball paradigm was conducted on an HP laptop (model 15-dw4143nia; 11th-generation Intel Core i7, 2.8 GHz, 8 GB RAM) running Windows 11. Pupillometry data were recorded online using Meye at a sampling frequency of 10 Hz.

For the naturalistic gaze-tracking experiment, both cameras were connected to a single Raspberry Pi 4, which ran a custom C++ script designed to receive control commands via a lab streaming layer (LSL) stream generated using PyLSL (version 1.17.6) (Kothe et al., 2025). Upon receiving start or stop commands, the script initiated or terminated video recording accordingly.

The LSL stream was generated on a separate computer using a custom Python script, which transmitted manual start and stop commands to control video acquisition remotely. Video recordings were acquired at 10 frames per second, corresponding to the maximum achievable frame rate given the hardware limitations of the Raspberry Pi when handling two simultaneous video streams. The Raspberry Pi was powered via USB-C using a portable power bank (YAGOPAL, KT-D009).

### Infrared illumination and optical-safety characterization

The infrared illumination used for eye-facing recordings was centered at 850 nm. To characterize ocular exposure under the configurations used in this study, we measured the radiant power of both the external infrared light source and the embedded camera LEDs at different source-to-detector distances. Measurements were obtained using a Molectron PowerMax 500AD laser power meter equipped with a thermal detector. The detector had a 2.5 cm diameter active area, corresponding to 4.91 cm^2^. Irradiance was estimated by dividing the measured radiant power by the detector active area. The maximum measured irradiance was 8.47 mW/cm^2^ for the external light source at 5 cm and 0.65 mW/cm^2^ for the embedded camera LEDs at 3 cm (Supplementary Figure 3). For a 10-min exposure, these values were below the anterior-eye infrared irradiance exposure limit reported in the ICNIRP guidelines for incoherent visible and infrared radiation (International Commission on Non-Ionizing Radiation Protection (ICNIRP), 2013). Experimental recordings were typically shorter than 10 min per run, with the external emitter positioned more than 50 cm from the eye and the embedded camera LEDs positioned approximately 4 cm or more from the eye. These measurements should be interpreted as an irradiance-based safety check rather than a formal photobiological safety certification. Users who modify the illumination hardware, reduce the source-to-eye distance, increase exposure duration, or deploy the system in pediatric, clinical, home-based, or unsupervised contexts should re-evaluate optical exposure for their specific configuration.

### Data analysis

Data analysis was performed using Python (3.8.18), NumPy version 1.26.4 (Harris et al., 2020), Pandas version 2.2.3 (McKinney, 2010), and SciPy version 1.13.1 (Virtanen et al., 2020). Data visualization was performed using MatPlotLib version 3.9.2 (Hunter, 2007) and Seaborn version 0.13.2 (Waskom, 2021). A summary of all the results is available in Supplementary Table 1.

## Results

We constructed a custom 3D-printed headset frame designed to support cameras for gaze tracking and pupillometry. The frame was produced using filament-based 3D printing and assembled with commonly available screws. It includes integrated channels for USB cable routing, a vertically adjustable nose rest, and temple arms whose position relative to the nose can be varied to modify the overall frame width. The ear supports are movable, allowing adaptation to different head shapes while improving stability during use. The eye-facing camera is mounted on a support connected via a ball joint, enabling multi-axis positioning. This support is attached to a linearly adjustable arm that permits adjustment of the camera’s distance from the eye (Fig. 1A). The combination of these features enables flexible positioning of the camera relative to the eye and facilitates alignment across users with different facial anatomies under the tested configurations.

The system supports multiple hardware configurations depending on the experimental application. It can be equipped with a single camera for monocular pupillometry or with two cameras for gaze tracking. The system is compatible with different camera models: a low-cost option without integrated infrared illumination requires external IR light sources, whereas a slightly more expensive option incorporates onboard infrared LEDs. The total hardware cost ranges from approximately €10 for the single camera configuration to around €17 for the dual camera configuration (Table 1). The cost estimate reported refers specifically to the minimal wearable MEYELens hardware required to assemble the 3D-printed frame and camera configuration. It does not include general laboratory infrastructure or reusable equipment. Because these components are specific to the experimental setup rather than to the wearable device itself, they may vary across applications and are often already available within standard laboratory infrastructure. For clarity, we therefore distinguish between the minimal wearable cost, which ranges from approximately €10 for the single-camera configuration to approximately €17 for the dual-camera configuration, and the cost of a complete operational setup. A detailed list of the additional components required to run all experiments reported in this manuscript is provided in Supplementary Table 2 and Supplementary Table 3.

**Table 1:** Detailed bill of materials for MEYELens minimal wearable hardware

Component	Listed purchase price (€)	Basis per wearable	Allocated cost per wearable (€)	Configuration	Source URL
PLA filament (Bambu Lab PLA Matte, black; 1 kg spool)	25.99	55 g from a 1 kg spool	1.43	Both configurations	Bambu Lab https://eu.store.bambulab.com/it/products/pla-matte?id=43992833261787
M3 screws and nuts kit	2.99	Approx. 1/7 kit	0.43	Both configurations	Amazon https://amzn.eu/d/30kOv5y
M2 screws and nuts kit	8.00	Approx. 1/10 kit	0.80	Both configurations	Amazon https://amzn.eu/d/2cbt0sm
USB camera with integrated infrared LEDs (eye-facing)	7.19	1 camera	7.19	One- and two-camera configurations	AliExpress https://www.aliexpress.com/item/1005004639261673.html
USB camera with integrated infrared LEDs (world-facing)	7.19	1 additional camera	7.19	Two-camera configuration only	AliExpress https://www.aliexpress.com/item/1005004639261673.html
Total: one-camera wearable			9.85	Pupillometry configuration	
Total: two-camera wearable			17.04	Gaze-mapping configuration	

### Pupillary light reflex

**Fig. 2:**
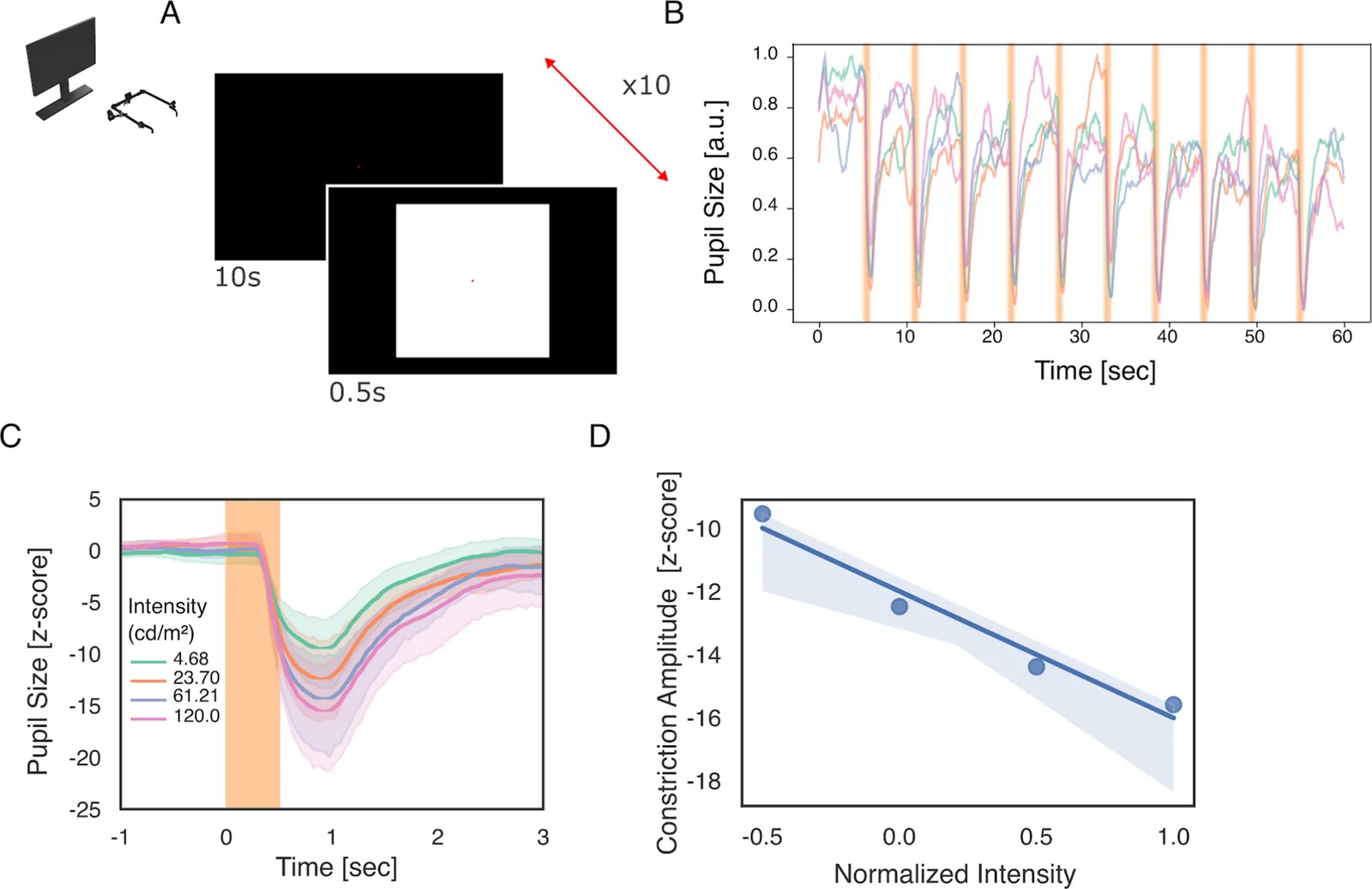
Pupillary light reflex (PLR) paradigm. **A** Stimulation sequence: a black screen with a central red fixation dot followed by a 0.5-s centrally presented square stimulus, repeated across trials. **B** Pupil size time course over the recordings, with stimulus epochs highlighted (*yellow bars*) and separate traces for each luminance condition. **C** Time-locked, *z*-scored pupil responses averaged across trials for each luminance condition. **D** Constriction amplitude as a function of normalized luminance intensity

We assessed the performance and sensitivity of our system using PLR (Fig. 2A), a well-established physiological response that reflects the functioning of both the visual pathway and the autonomic nervous system activity (Kelbsch et al., 2019; McDougal & Gamlin, 2015). Flash onset elicited a robust pupil constriction, evident as a transient decrease in pupil area (Fig. 2B). This response was present at all tested luminance levels. When normalized and averaged within each condition, pupil constriction amplitude scaled with luminance, with brighter flashes producing larger constrictions (Fig. 2C). To quantify this effect (Ellis, 1981), we computed the Pearson correlation between luminance and the average constriction amplitude. This analysis revealed a strong inverse relationship (Pearson correlation, r = − 0.98, *r*^2^ = 0.96, *p* < 0.05), with higher luminance associated with larger pupil constrictions (Fig. 2D). Together, these results indicate that MEYELens was able to detect graded PLR responses across light intensities under the tested conditions.

### Pupil frequency tagging

**Fig. 3:**
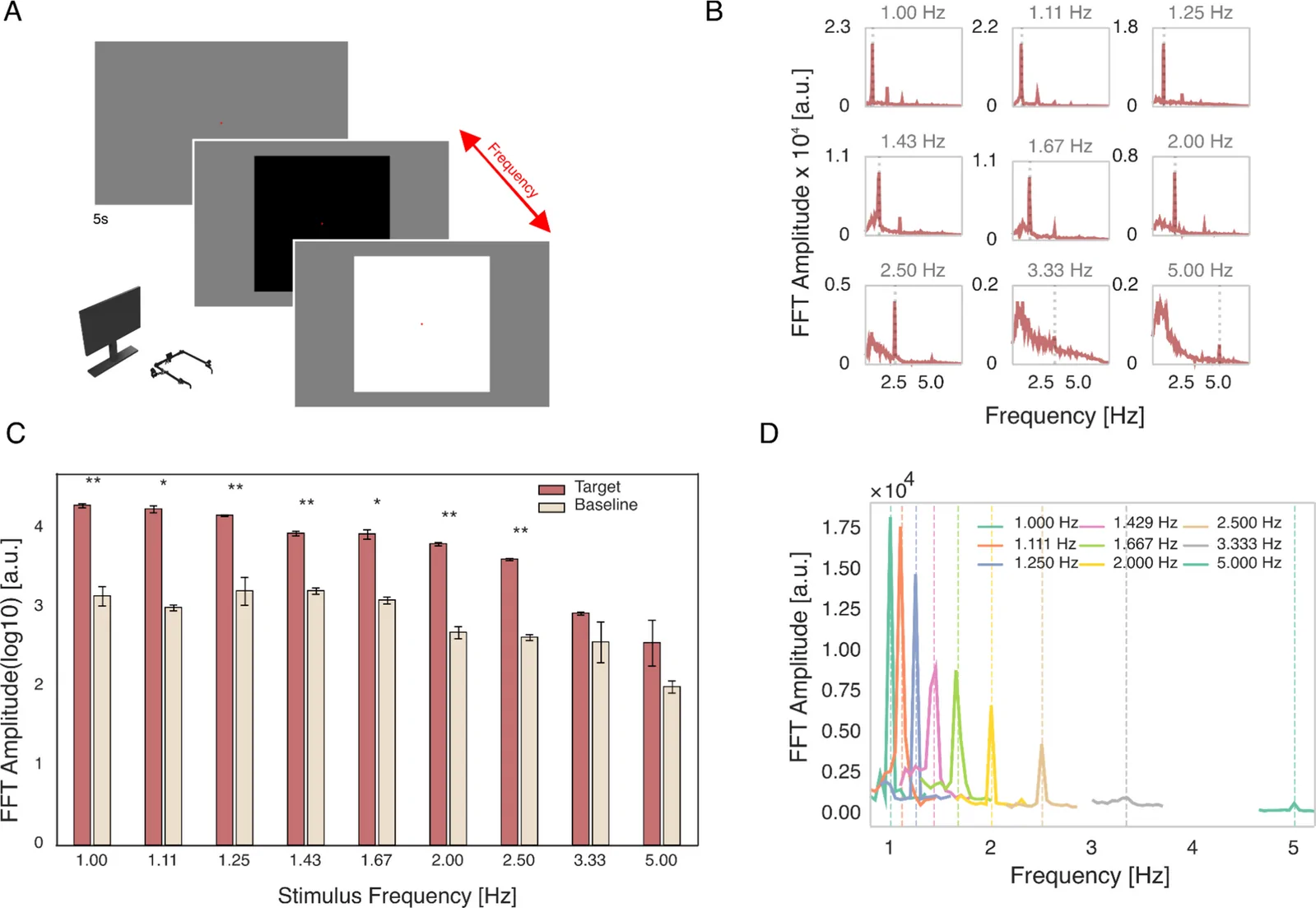
Pupil frequency tagging paradigm. **A** Stimulation sequence: a central fixation point with a black–white square stimulus flickering at a fixed frequency, repeated across multiple target frequencies. **B** Mean FFT amplitude spectra for each stimulation frequency, with the *dashed vertical line* indicating the driving (target) frequency. **C** Bar plot of mean FFT amplitude (log10) computed in a narrow band centered on the target frequency compared with neighboring frequency bins, shown across stimulation frequencies. **D** Overlaid mean FFT spectra for all stimulation frequencies, with *colored dashed vertical lines* marking each target frequency. (*: *p *< 0.05. **:* p *< 0.01, ***:* p *< 0.001)

To assess the temporal resolution and responsiveness of our system, we used a PFT paradigm in which three participants were presented with flickering black-and-white stimuli at various fixed frequencies (Fig. 3A). The pupil tends to respond rhythmically when exposed to flickering light, producing changes at the same frequency as the stimulus (Lorenceau et al., 2024; Naber et al., 2013a, b).

We performed a fast Fourier transform (FFT) on the pupil area time series for each trial and averaged the resulting power spectra across the three participants. Across most stimulation frequencies, the pupil signal showed a clear spectral peak at the driving frequency (Fig. 3B), consistent with strong entrainment.

To quantify both response strength and frequency selectivity, we compared the mean spectral amplitude in a narrow band centered on the stimulation frequency with the mean amplitude in neighboring frequency bins (Fig. 3C). Across frequencies, target amplitude was significantly higher than surround amplitude (repeated-measures ANOVA, main effect of type: *F*(1,2) = 737.79, *p* = 0.00135, ηp^2^ = 0.997), indicating robust frequency-locked responses. The magnitude of this target–surround difference varied across stimulation frequencies (type × frequency interaction: *F*(8,16) = 50.15, *p* = 6.96 × 10⁻^1^⁰, ηp^2^ = 0.962). Consistent with this, follow-up paired comparisons showed significant target > surround differences for most frequencies in the 1.0–2.5 Hz range (all *p* < 0.05), whereas higher frequencies did not always reach significance despite effects in the expected direction.

These findings indicate that, under the tested conditions, MEYELens was able to detect frequency-specific pupil responses consistent with frequency-tagging paradigms.

### Oddball paradigm

**Fig. 4:**
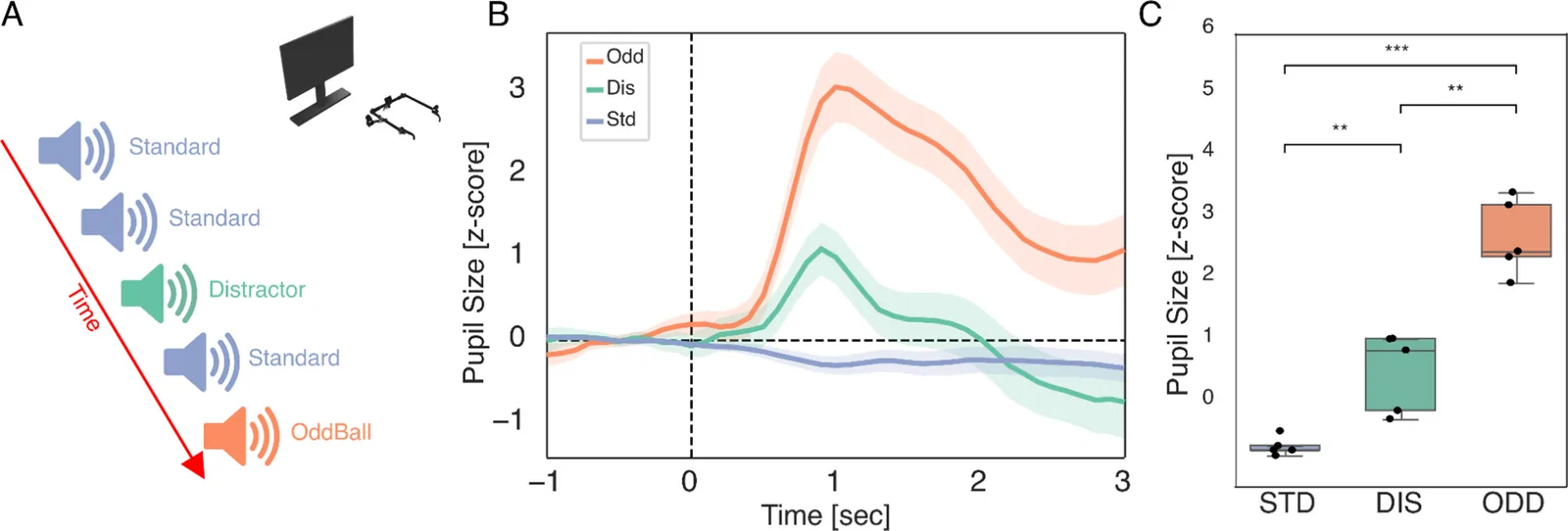
Auditory oddball paradigm. **A** Auditory sequence showing frequent standard tones, rare distractors, and rare oddball targets presented over time. **B** *Z*-scored, time-locked mean pupil responses for standard (Std), distractor (Dis), and oddball (odd) tones, aligned to stimulus onset (*vertical dashed line*). **C **Boxplot of mean peak* z*-scored pupil responses within the post-stimulus analysis window for each condition. (*: *p *< 0.05. **:* p *< 0.01, ***:* p *< 0.001)

To evaluate whether the system could detect cognitively driven pupillary responses, we implemented an auditory oddball task. The stimulus sequence included a frequent standard tone (440 Hz; 90% of trials), a rare distractor tone (220 Hz; 5%), and a rare oddball target tone (880 Hz; 5%). Participants (*n* = 5) were instructed to respond with a keyboard press only to the oddball tone (Fig. 4A). The *z*-scored pupil time series from the subjects were averaged across trials and participants for each condition. A repeated-measures ANOVA revealed a significant main effect of condition (standard, distractor, oddball) on the response (repeated-measures ANOVA, *F* = 48.26, *p* < 0.001). Post-hoc paired *t* tests showed significant differences for all pairwise comparisons: standard vs. distractor (paired *t* test, *t* = – 4.7, *p* < 0.01); standard vs. oddball (paired *t* test, *t* = – 10.9, *p* < 0.001); distractor vs. oddball (paired *t* test, *t* = – 4.8, *p* < 0.01). These results (Fig. 4B–C) are consistent with previous auditory oddball findings (Liao et al., 2016; Strauch et al., 2020) and suggest that MEYELens can capture cognitively driven pupillary responses under the tested conditions.

### Parallel PLR

**Fig. 5:**
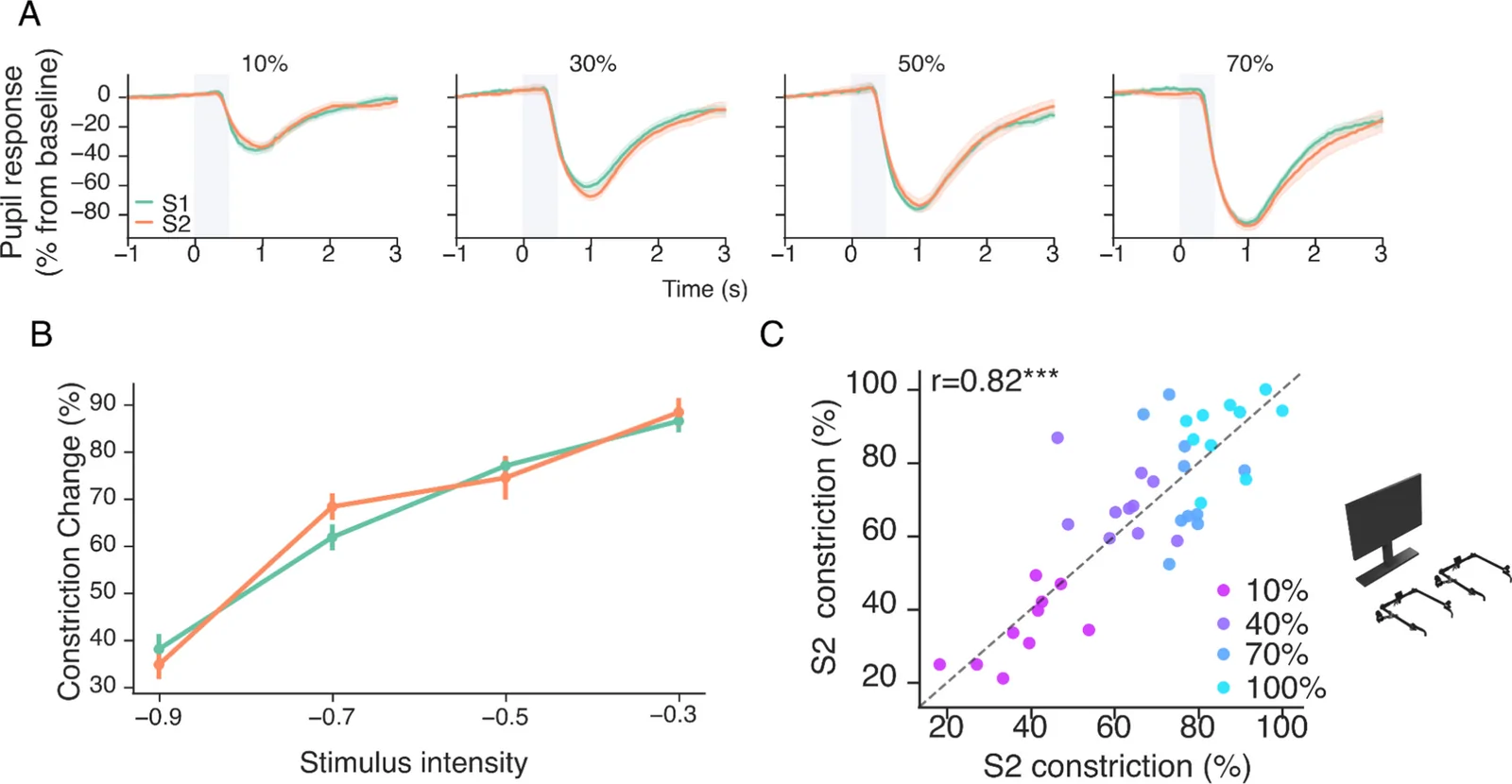
Parallel pupillary light reflex recordings from two participants. **A** Baseline-normalized pupil responses over time for two subjects (S1, S2) at multiple stimulus intensities, with the stimulus period indicated by the *shaded window*. (**B**) Mean constriction change (%) as a function of stimulus intensity for each subject. **C **Scatter plot comparing constriction magnitude between the two subjects across intensities. (*: *p *< 0.05. **:* p *< 0.01, ***:* p *< 0.001)

To demonstrate the flexibility and scalability of the system, we tested the pupillary light reflex paradigm in parallel on two participants (pPLR). In this configuration, both participants wore the pupillometry wearable system and viewed the same light stimulus presented on a shared monitor. The pupil diameter time courses recorded from the two individuals showed highly similar trajectories over the course of the stimulus sequence and of the various intensities (Fig. 5A–B). When we quantified this similarity by computing the Pearson correlation between the constrictions at different intensities, we found a strong positive correlation (Pearson correlation, *r* = 0.86, *r*^2^ = 0.74, *p* < 0.001), indicating a high degree of agreement in the pupillary responses (Fig. 5C). The successful acquisition of pupil data in parallel from two subjects demonstrates that the system can accommodate multiple users without apparent degradation in signal quality or synchronization. This suggests that the system is technically capable of supporting parallel data collection, highlighting its potential scalability for multi-participant experimental setups.

### Independent gaze-calibration validation

To estimate gaze-prediction error, we used a held-out validation procedure. Across the 1200 held-out validation samples, the mean angular error was 1.20°, the median error was 1.11°, and the RMSE was 1.32°. Because each validation target was sampled 60 times, we also summarized performance separately for each of the 20 validation positions. As a separate check, the five original calibration positions were presented again immediately after calibration, without refitting or updating the model; this retest yielded a median error of 0.46°. Eighteen validation samples, corresponding to 1.5% of the dataset, were classified as blink and were linearly interpolated.

### Comparison with a commercial eye-tracking system

**Fig. 6:**
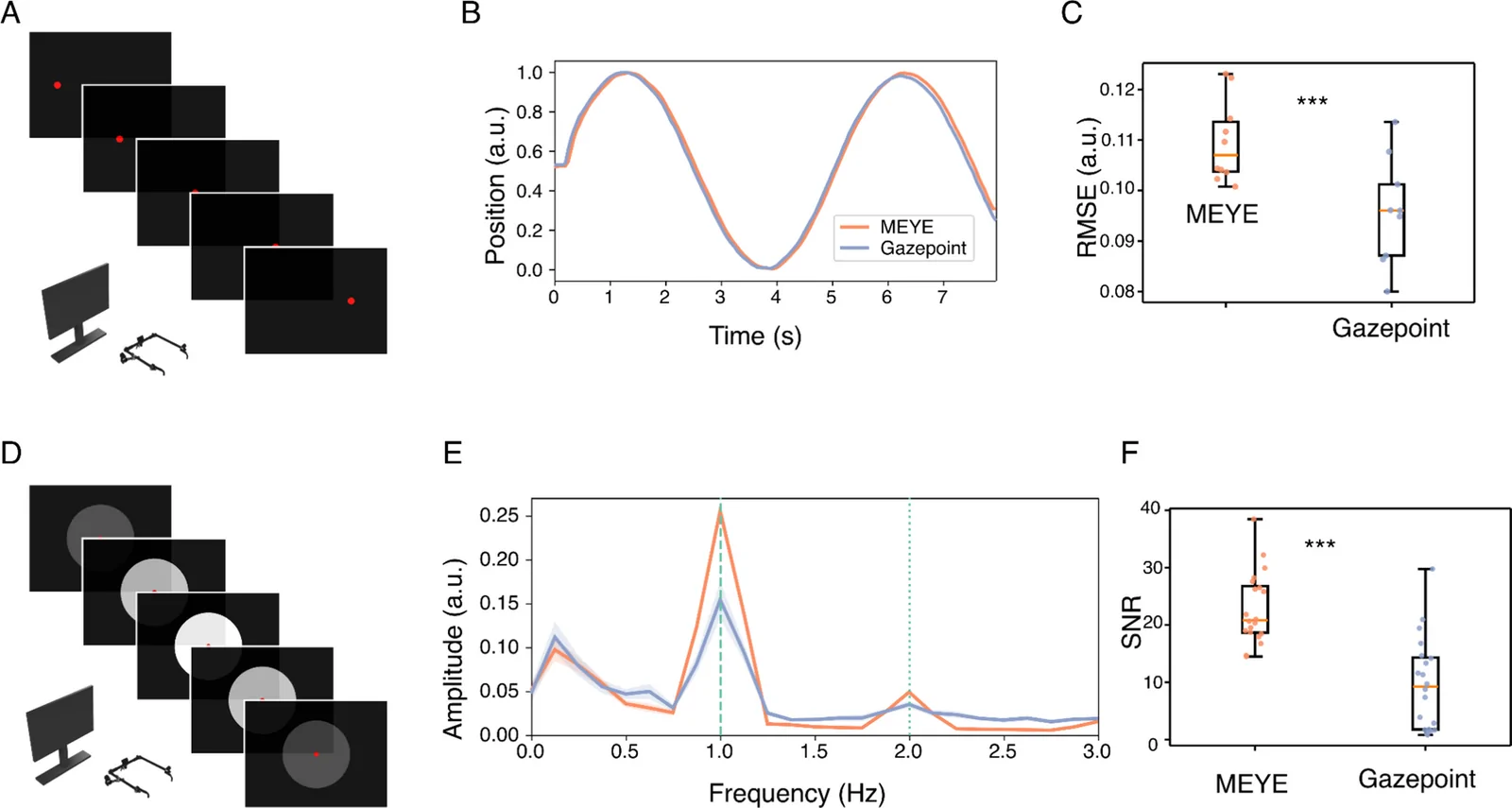
Comparison between MEYE Lens and a commercial eye tracker (Gazepoint GP3) in smooth pursuit and pupil frequency tagging. **A** Smooth-pursuit stimulus: a red dot moving along a sinusoidal trajectory across the screen. **B** Mean horizontal gaze position traces over time recorded with MEYE Lens and Gazepoint during smooth pursuit. **C** Boxplot comparison of smooth-pursuit root mean square error (RMSE) for the two systems across trials. **D** Pupil frequency-tagging stimulus: a central flickering luminance patch presented at a fixed frequency. **E** Mean FFT amplitude spectra from MEYE Lens and Gazepoint during tagging, with vertical markers at the stimulation frequency (1 Hz) and its first harmonic (2 Hz). **F** Boxplot comparison of signal-to-noise ratio (SNR) at the tagged frequency for MEYE Lens and Gazepoint. (*: *p *< 0.05. **:* p *< 0.01, ***:* p *< 0.001)

To evaluate tracking accuracy during continuous gaze behavior, we performed a direct comparison between our system and a commercial eye tracker (GP3) using two established paradigms: smooth pursuit and PFT. In the smooth-pursuit task, the participant was instructed to track a red dot moving along a horizontal sinusoidal trajectory (Fig. 6A). Although the trial-averaged horizontal gaze position traces from MEYELens and GP3 showed qualitatively similar waveforms (Fig. 6B), the systems differed significantly in accuracy, as reflected by RMSE. To quantify accuracy relative to the nominal target trajectory, we fit a 0.2-Hz sinusoid to the horizontal gaze position on each trial and computed the root-mean-square error (RMSE; Fig. 6C). The average RMSE distributions differed significantly between systems, with GP3 exhibiting lower RMSE (paired *t* test, *t* = 5.7, *p* < 0.001), indicating that MEYELens has a lower gaze-tracking accuracy. In the PFT task, the participant maintained fixation on a central red dot while a background circle flashed at 1 Hz (Fig. 6D). For both systems, the mean FFT amplitude spectra showed a prominent peak at the stimulation frequency (1 Hz) and a second harmonic at 2 Hz (Fig. 6E), consistent with frequency-specific pupil modulation. Response magnitude at 1 Hz was larger for MEYELens (0.255 ± 0.033) than for GP3 (0.154 ± 0.093). We quantified spectral quality using a signal-to-noise ratio (SNR) defined as the amplitude of the FFT bin closest to 1 Hz divided by the median amplitude of bins in the 0.3–3 Hz band, excluding bins within ± 2 frequency bins around 1 Hz and its harmonics. Trial-wise paired statistics showed that MEYELens achieved significantly higher SNR than GP3 (paired *t* test, *t* = 6.2, *p* < 0.001; Fig. 6F). Thus, while both systems captured the tagged response, MEYELens produced a stronger and cleaner spectral peak. Overall, under the tested conditions, MEYELens demonstrated lower gaze-tracking accuracy than the commercial device in smooth pursuit, but higher pupillometry SNR in the PFT paradigm.

### Naturalistic gaze-tracking

**Fig. 7:**
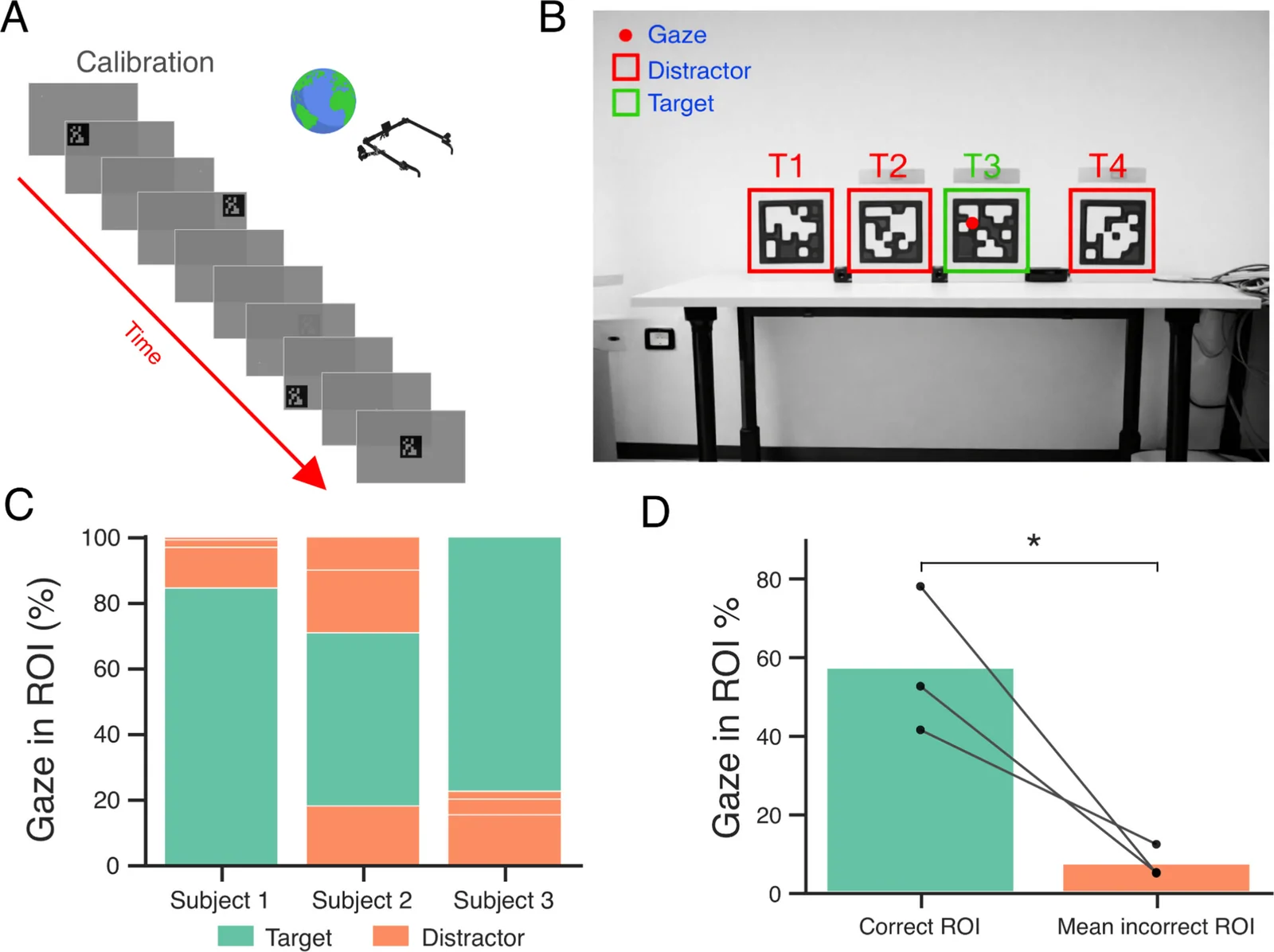
Naturalistic sound-localization task and gaze-to-target quantification. **A** Calibration sequence used in the portable setup, shown as successive fixation targets over time. **B** Example scene view with four regions of interest (ROIs) corresponding to the boxes; the detected gaze point is overlaid and the target ROI is highlighted. **C** Proportion of gaze samples falling within the target ROI versus distractor ROIs for each participant. **D** Group-level comparison of gaze samples in the correct ROI versus the mean of incorrect ROIs. (*: *p *< 0.05. **:* p *< 0.01, ***:*p *< 0.001)

To evaluate the performance of the portable configuration in a naturalistic and attentional task, we designed a simple spatial localization paradigm in which participants responded to an auditory cue. In each trial, after calibrating the gaze to the world-facing camera (Fig. 7A), a phone rang from behind one of four identical boxes positioned in front of the participant while we simultaneously recorded eye data (Fig. 7B); recordings were then processed offline after the experiment. Because overt spatial orienting is typically accompanied by coordinated shifts of attention and gaze (Braga et al., 2016; Pomper & Chait, 2017), we expected participants to orient their gaze toward the sound source. This paradigm allowed us to test whether the system could detect such stimulus-driven gaze behavior in an unconstrained, freely moving context. Gaze data confirmed that participants consistently directed their attention and gaze toward the correct location. Across all three participants, the number of gaze samples falling within the region corresponding to the target box was higher than the number for the three non-target boxes (Fig. 7C). A paired *t* test on the average results across subjects showed that the proportion of gaze samples directed at the target box was significantly higher (paired *t* test,* t* = 4.8, *p* < 0.05) than that directed at the non-target boxes, supporting the presence of spatial attentional bias (Fig. 7D). These findings suggest that, under the tested conditions, the portable system was able to capture spatially selective gaze responses in an unconstrained, attention-driven task while allowing free movement within a natural setting.

## Discussion

In this work, we present MEYELens, an open-source, fully 3D-printable, and cost-effective wearable system for pupillometry and gaze tracking. The system is designed to be modular, customizable, and reproducible, providing a practical alternative to commercial eye-tracking solutions that are often limited by high costs and proprietary constraints (Casas & Chandrasekaran, 2019; Toivanen et al., 2017; Zandi et al., 2021). By substantially lowering the financial barrier associated with gaze-tracking technology, MEYELens expands access to tools that are increasingly important in cognitive neuroscience, psychology, and human--computer interaction research.

The primary limitation of MEYELens is its relatively low frame rate of approximately 10–20 Hz. While this sampling rate is sufficient for many cognitive and physiological paradigms, it is not suitable for capturing rapid eye movements such as microsaccades (Poletti & Rucci, 2016). This limitation stems from the use of low-cost camera modules. However, the modular design of the system allows straightforward upgrades as higher-frame-rate affordable cameras become available, without requiring substantial redesign.

MEYELens supports diverse experimental paradigms and can be adapted to different research contexts and anatomical characteristics. Both the hardware design and the software pipeline for data acquisition and processing are open-source, enabling replication, modification, and extension by end users. This flexibility allows researchers to integrate additional sensors, customize experimental workflows, and interface with external devices or data streams, enabling a broad range of scientific applications.

Beyond its research applications, MEYELens also serves as an effective educational platform. It offers hands-on exposure to 3D design, hardware prototyping, programming, and experimental methodology. Its modular architecture encourages interdisciplinary learning and makes it well suited for teaching laboratories, training courses, and workshops. The simplicity of the system and ease of assembly further lower the barrier for students and early-career researchers to engage with eye-tracking methods.

Despite its low cost, MEYELens showed promising performance across the feasibility experiments reported here. The system was able to capture key pupillary responses, including luminance-driven constriction during the PLR, frequency-specific modulation in PFT paradigms, and stimulus-dependent dilation in oddball tasks. These results were obtained using modest hardware, indicating that useful pupillometric and gaze-related measurements can be obtained under constrained computational conditions. Overall, in this single-participant comparison, MEYELens showed higher smooth-pursuit gaze error than the commercial device, as reflected by a significantly higher RMSE. Importantly, this reduction in accuracy did not alter the qualitative structure of the gaze trajectories, which closely followed the target motion in both systems. This suggests that, for experimental paradigms relying on relative gaze position, temporal alignment, or coarse trajectory tracking, MEYELens may be suitable, although higher-precision gaze-tracking applications remain better served by dedicated commercial systems. Conversely, in the frequency-tagging paradigm, MEYELens produced a higher pupillometry signal-to-noise ratio than the commercial system, resulting in a more pronounced spectral peak at the stimulation frequency. This advantage is experimentally meaningful, as higher SNR directly improves the detectability and robustness of tagged responses. Thus, while MEYELens exhibits reduced spatial accuracy in continuous gaze tracking, the present results suggest that it can provide useful pupillometric signal quality for frequency-based analyses under the tested conditions. The present study should therefore be interpreted as a feasibility demonstration rather than as a comprehensive validation across users, devices, and settings. Future work should assess test–retest reliability, inter-subject variability, variability across independently printed devices, and robustness to facial anatomy, glasses, eyelashes, iris color, motion, age, and clinical status, particularly before deployment in pediatric, clinical, home-based, or unsupervised contexts.

MEYELens also shows potential for parallel data collection. The pPLR experiment suggested that multiple systems can operate simultaneously under the tested conditions without apparent interference. Crucially, the system is wearable, lightweight, and can be assembled into a compact, self-contained setup that runs on commonly available hardware (laptops or Raspberry Pi boards) and can be powered via portable battery packs. This combination of low cost, reproducibility, and minimal infrastructure requirements may facilitate future deployment in field studies, classrooms, clinical environments, and other space- or resource-limited contexts where conventional eye-tracking systems are impractical. By providing a fully open and low-cost solution, MEYELens contributes to reducing geographical and economic disparities in access to advanced research tools. Its affordability and reproducibility may help underfunded institutions and researchers in low- and middle-income countries to conduct pupillometry and gaze-tracking studies, promoting greater inclusivity and diversity in scientific research.

## Supplementary Information

Below is the link to the electronic supplementary material.

Supplementary file1 (PDF 1216 KB)

Supplementary file2 (PDF 76 KB)

Supplementary file3 (PDF 21 KB)

Supplementary file4 (PDF 29 KB)

## Data Availability

The original data and materials are publicly available from: https://zenodo.org/records/18429496. The 3 d models are publicly available from: https://3d.nih.gov/entries/3DPX-023297. https://github.com/raffaelemazziotti/MEYElens/tree/main/3d_print_files. https://makerworld.com/en/models/2267867-meyelens-3d-printed-pupillometry-gaze-tracking.

## Code availability

The code library is publicly available from: https://github.com/raffaelemazziotti/MEYElens.

In addition, a dedicated project website is available at https://meyelens.com. The website serves as a centralized resource for the MEYELens platform and provides software installation instructions, usage documentation, hardware information, bill of materials, 3D model resources, tutorials, troubleshooting guidance, and links to the associated repositories and downloadable materials.
