# Supplementary material for: MEYELens: An affordable, open-source, 3D-printable eyewear platform for pupillometry and gaze tracking
Source: Behav Res Methods. 2026 Aug 21;58(10):277. doi: 10.3758/s13428-026-03157-z (PMC13498615; doi:10.3758/s13428-026-03157-z)
Supplement: Supplementary file 1 [file 13428_2026_3157_MOESM1_ESM.pdf]

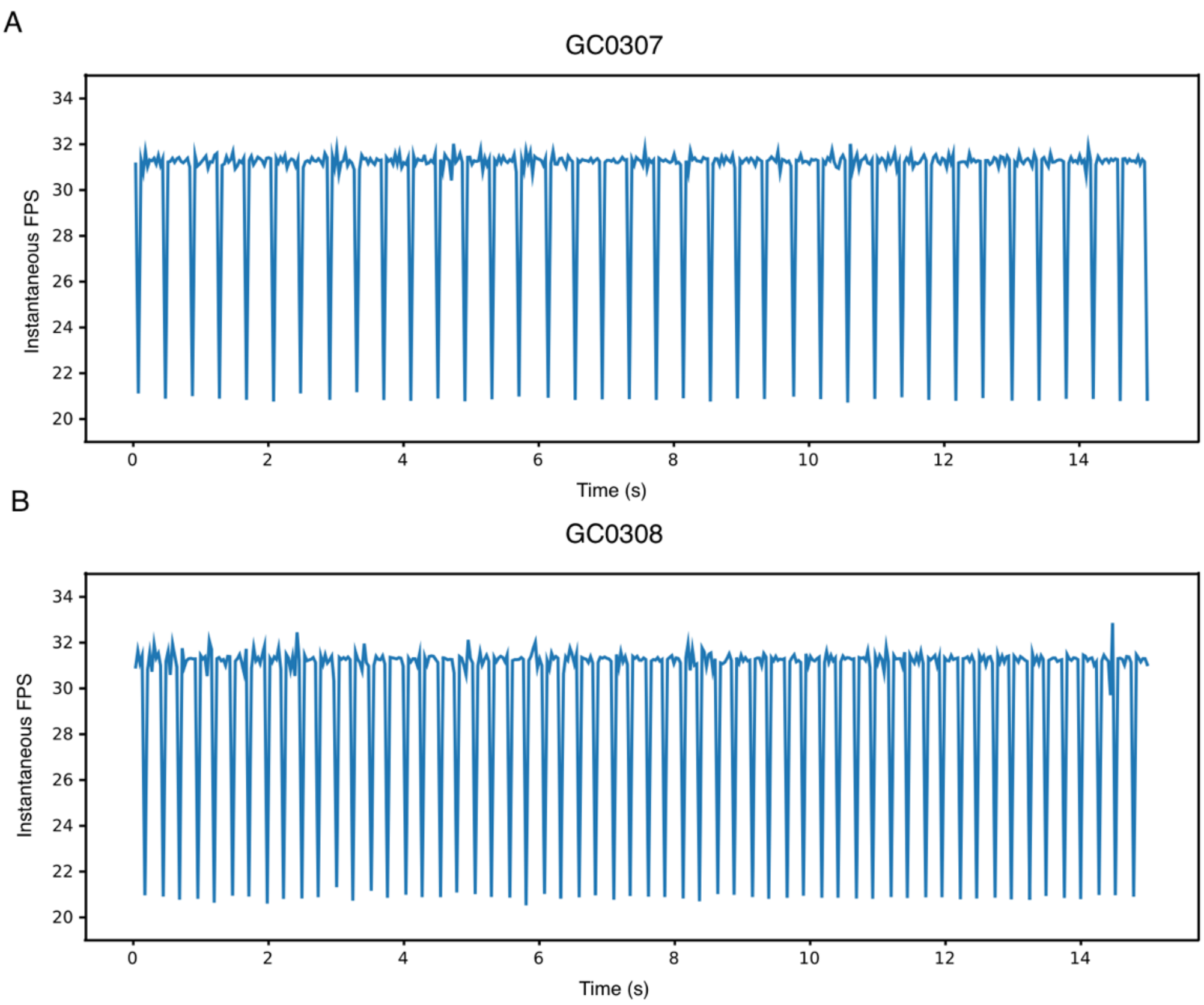

Supplementary Figure 1. Instantaneous FPS for A the GC0307 camera and B the GC0308 camera

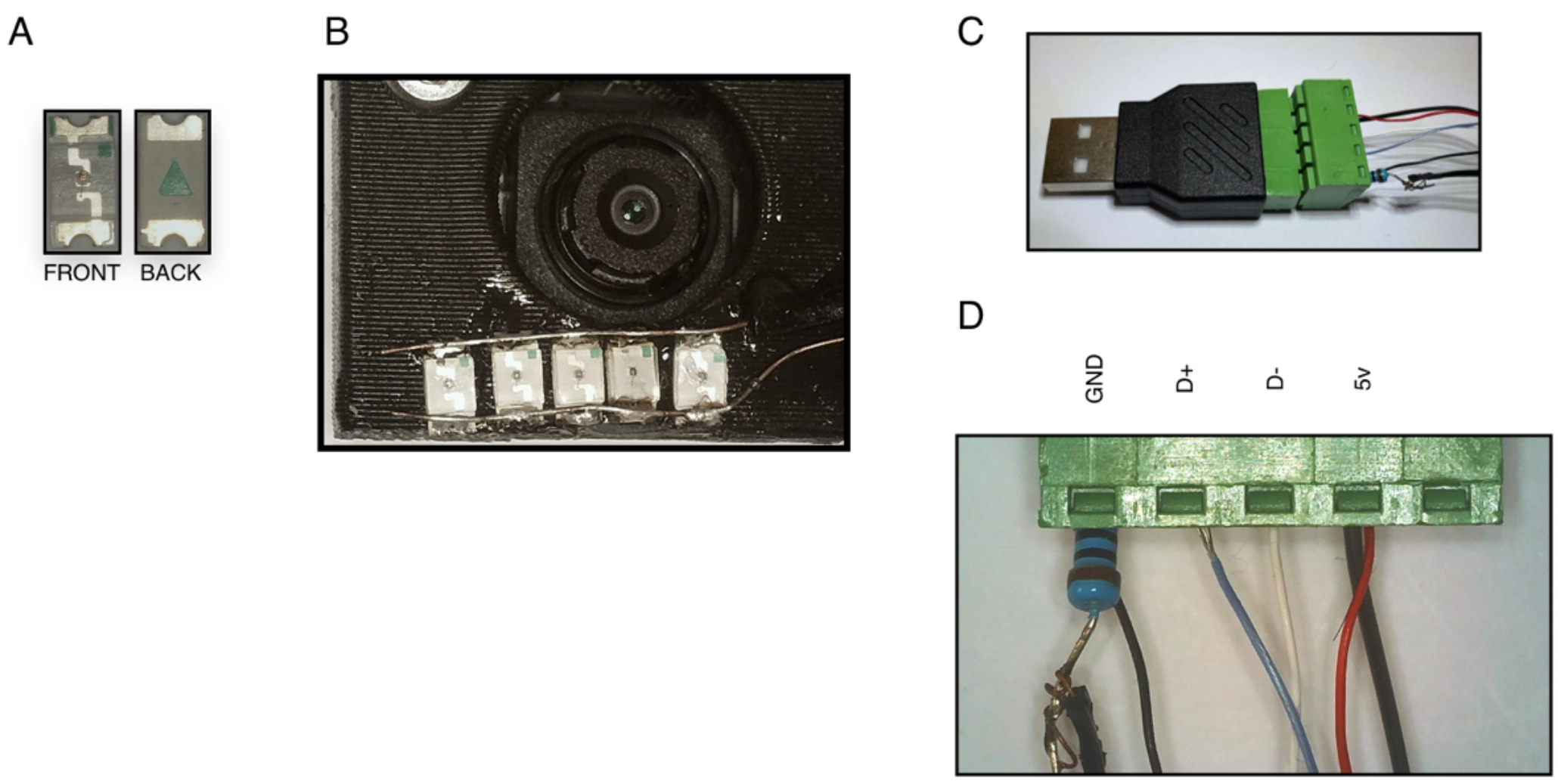

Supplementary Figure 2. The optional IR LED setup

(A) The LED used.

(B) The LED mounted on the camera mount.

(C) The connector used to power both the led and the camera with one USB connection.

(D) The connections for the LED array (on GND and 5V with a resistance of 20 Ω) and the camera.

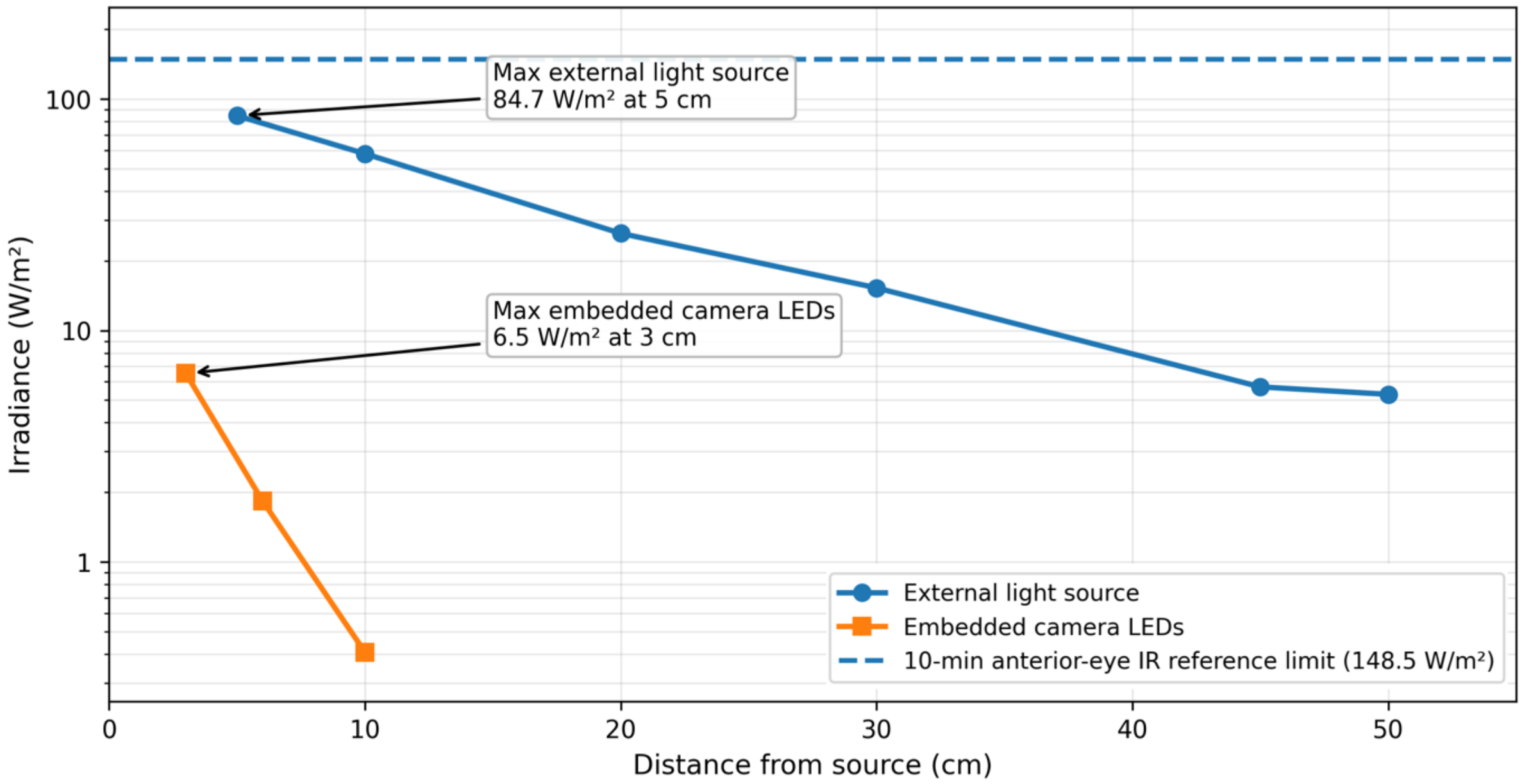

Supplementary Figure 3. Distance-dependent irradiance of the 850nm infrared illumination sources
