# Supplementary material for: MEYELens: An affordable, open-source, 3D-printable eyewear platform for pupillometry and gaze tracking
Source: Behav Res Methods. 2026 Aug 21;58(10):277. doi: 10.3758/s13428-026-03157-z (PMC13498615; doi:10.3758/s13428-026-03157-z)
Supplement: Supplementary file 2 [file 13428_2026_3157_MOESM2_ESM.pdf]

Figure	Comparison	Metric	Test	N	N1	N2	Statistic	Value	df	p	Effect	Effect value	95% CI low	95% CI high
Figure 2D	Intensity vs amplitude	Avg per-trial minimum (z)	Pearson correlation	4			r	-0.982	2	.018				
Figure 3C	1.0000 Hz	FFT amplitude	Paired t-test	3			t	22.01	2	.002	Cohen's dz	12.707	14865.089	22089.169
Figure 3C	1.1111 Hz	FFT amplitude	Paired t-test	3			t	8.913	2	.012	Cohen's dz	5.146	8844.788	25352.564
Figure 3C	1.2500 Hz	FFT amplitude	Paired t-test	3			t	12.133	2	.007	Cohen's dz	7.005	8394.859	17620.711
Figure 3C	1.4286 Hz	FFT amplitude	Paired t-test	3			t	10.457	2	.009	Cohen's dz	6.037	4248.695	10189.853
Figure 3C	1.6667 Hz	FFT amplitude	Paired t-test	3			t	5.995	2	.027	Cohen's dz	3.461	2126.275	12937.278
Figure 3C	2.0000 Hz	FFT amplitude	Paired t-test	3			t	16.052	2	.004	Cohen's dz	9.267	4412.596	7644.521
Figure 3C	2.5000 Hz	FFT amplitude	Paired t-test	3			t	26.899	2	.001	Cohen's dz	15.53	3138.093	4333.172
Figure 3C	3.3333 Hz	FFT amplitude	Paired t-test	3			t	0.991	2	.426	Cohen's dz	0.572	-1115.861	1783.837
Figure 3C	5.0000 Hz	FFT amplitude	Paired t-test	3			t	1.671	2	.237	Cohen's dz	0.965	-666.09	1511.916
Figure 3C	Frequency (main effect)	FFT amplitude	Repeated-measures ANOVA	3			F	925,872	8, 16	< .001	partial eta squared	978,855		
Figure 3C	Target vs Surround (main effect)	FFT amplitude	Repeated-measures ANOVA	3			F	737,789	1, 2	1,353	partial eta squared	997,297		
Figure 3C	Type × Frequency (interaction)	FFT amplitude	Repeated-measures ANOVA	3			F	5,014,789	8, 16	< .001	partial eta squared	961,648		
Figure 4C	Standard vs Distractor	Peak	Paired t-test	5	5	5	t	-4.689	4	.009	Cohen's dz	-2.097	-1.934	-0.495
Figure 4C	Distractor vs Oddball	Peak	Paired t-test	5	5	5	t	-4.826	4	.008	Cohen's dz	-2.158	-3.412	-0.92
Figure 4C	Standard vs Oddball	Peak	Paired t-test	5	5	5	t	-10.905	4	< .001	Cohen's dz	-4.877	-4.241	-2.52
Figure 4C	Standard, Distractor, Oddball	Peak	Repeated-measures ANOVA	5			F	48.256	2, 8	< .001	generalized eta squared	0.9		
Figure 5C	Subject 1 vs Subject 2	Peak constriction (%)	Pearson correlation	40			r	0.816	38	< .001				
Gaze validation	Held-out gaze	Angular error	Mean	1200			Mean	1.2						
Gaze validation	Held-out gaze	Angular error	Median	1200			Median	1.11						
Gaze validation	Held-out gaze	Angular error	RMSE	1200			RMSE	1.32						
Figure 6C	MEYE vs Gazepoint	RMSE	Paired t-test	9	9	9	t	5.735	8	< .001	Cohen's dz	1.912	0.009	0.02
Figure 6F	MEYE vs Gazepoint	SNR	Paired t-test	20	20	20	t	6.19	19	< .001	Cohen's dz	1.384	8.773	17.736
Figure 7D	Correct vs Incorrect	Gaze in ROI (%)	Paired t-test	3	3	3	t	4.853	2	.040	Cohen's dz	2.802	7.041	117.194

Supplementary Table 1. Statistical analyses of the experimental results.
