# Supplementary material for: MEYELens: An affordable, open-source, 3D-printable eyewear platform for pupillometry and gaze tracking
Source: Behav Res Methods. 2026 Aug 21;58(10):277. doi: 10.3758/s13428-026-03157-z (PMC13498615; doi:10.3758/s13428-026-03157-z)
Supplement: Supplementary file 3 [file 13428_2026_3157_MOESM3_ESM.pdf]

Item	Example model / cost basis	Category	Source URL	Listed price (€)
One-camera MEYELens	PLA, M2/M3 fasteners, and one integrated-IR camera	Wearable	See Table 1	9.85
Two-camera MEYELens	Two-cameras	Wearable	See Table 1	17.04
3D printer	Bambu Lab A1	Fabrication equipment	https://bambulab.com/en	249.00
UV-curing adhesive	One tube; multiple builds	Fabrication consumable	https://amzn.eu/d/053iQqxH	9.99
External IR	One illuminator	Recording accessory	https://amzn.eu/d/0gmQ0qnX	33.00
USB extension cable	Single-device extension	Recording accessory	https://amzn.eu/d/03ZkeQgd	6.99
Dual USB extension cable	Two-device extension	Recording accessory	https://amzn.eu/d/0h2XTN7q	11.99
USB-C hub	USB-C host adapter	Host accessory	https://amzn.eu/d/06sX07I5	12.22
Laptop computer	Apple 13-inch MacBook Air	Host computer	www.apple.com	1249.00
Portable host	Raspberry Pi	Host computer	https://amzn.eu/d/07fTJTqJ	50.00
Power bank	Portable USB power source	Power accessory	https://amzn.eu/d/0ism99rf	22.00
Calibratable display	ASUS ProArt PA278CGV	Display equipment	https://www.asus.com/displays-desktops/monitors/all-series/	331.99
Display colorimeter	Datacolor Spyder	Calibration equipment	https://www.photospecialist.it/datacolor-spyder	189.00
Headphones	General-purpose wired headphones	Task accessory	https://amzn.eu/d/0fQFLPjK	12.99
Chin and head rest	Low-cost research-grade DIY allowance	Task accessory	https://niall.phd/pdfs/samaha_cheapchinrest.pdf	100.00

Supplementary Table 2. Bill of materials for the MEYELens wearable and complete operational setup.
