# Supplementary material for: MEYELens: An affordable, open-source, 3D-printable eyewear platform for pupillometry and gaze tracking
Source: Behav Res Methods. 2026 Aug 21;58(10):277. doi: 10.3758/s13428-026-03157-z (PMC13498615; doi:10.3758/s13428-026-03157-z)
Supplement: Supplementary file 4 [file 13428_2026_3157_MOESM4_ESM.pdf]

Operational setup (included items)	Estimated total (€)
Tethered one-camera setup (one-camera wearable; 3D printer; adhesive; USB extension cable; USB-C hub; laptop host)	1537.05
Tethered two-camera setup (two-camera wearable; 3D printer; adhesive; dual USB cable; USB-C hub; laptop host)	1549.24
Portable two-camera setup (two-camera wearable; 3D printer; adhesive; dual USB cable; Raspberry Pi host; power bank)	360.02
Full laboratory setup (tethered two-camera setup; calibratable display; colorimeter; headphones; chin/head rest)	2183.22
**Comprehensive all-capabilities inventory (full laboratory setup; external IR illuminator; Raspberry Pi host; power bank)**	**2288.22**

Supplementary Table 3. Estimated costs of representative MEYELens operational setups.
